# Understanding the influence of teacher–learner relationships on learners’ assessment perception

**DOI:** 10.1007/s10459-019-09935-z

**Published:** 2019-10-29

**Authors:** Suzanne Schut, Jan van Tartwijk, Erik Driessen, Cees van der Vleuten, Sylvia Heeneman

**Affiliations:** 1 Faculty of Health, Medicine and Life Sciences, School of Health Professions Education, Maastricht University, Maastricht, The Netherlands; 2 Department of Educational Development and Research, Faculty of Health, Medicine and Life Sciences, Maastricht University, Universiteitssingel 60, 6229 ER Maastricht, The Netherlands; 3 Department of Education, Utrecht University, Utrecht, The Netherlands; 4 Department of Pathology, Faculty of Health, Medicine and Life Sciences, Maastricht University, Maastricht, The Netherlands

**Keywords:** Assessment for learning, Low-stake assessments, Teacher–learner relationships, Faculty development

## Abstract

Low-stakes assessments are theorised to stimulate and support self-regulated learning. They are feedback-, not decision-oriented, and should hold little consequences to a learner based on their performance. The use of low-stakes assessment as a learning opportunity requires an environment in which continuous improvement is encouraged. This may be hindered by learners’ perceptions of assessment as high-stakes. Teachers play a key role in learners’ assessment perceptions. By investigating assessment perceptions through an interpersonal theory-based perspective of teacher–learner relationships, we aim to better understand the mechanisms explaining the relationship between assessment and learning within medical education. First, twenty-six purposefully selected learners, ranging from undergraduates to postgraduates in five different settings of programmatic assessment, were interviewed about their assessment task perception. Next, we conducted a focussed analysis using sensitising concepts from interpersonal theory to elucidate the influence of the teacher–learner relationship on learners’ assessment perceptions. The study showed a strong relation between learners’ perceptions of the teacher–learner relationship and their assessment task perception. Two important sources for the perception of teachers’ agency emerged from the data: positional agency and expert agency. Together with teacher’s communion level, both types of teachers’ agency are important for understanding learners’ assessment perceptions. High levels of teacher communion had a positive impact on the perception of assessment for learning, in particular in relations in which teachers’ agency was less dominantly exercised. When teachers exercised these sources of agency dominantly, learners felt inferior to their teachers, which could hinder the learning opportunity. To utilise the learning potential of low-stakes assessment, teachers are required to stimulate learner agency in safe and trusting assessment relationships, while carefully considering the influence of their own agency on learners’ assessment perceptions. Interpersonal theory offers a useful lens for understanding assessment relationships. The Interpersonal Circumplex provides opportunities for faculty development that help teachers develop positive and productive relationships with learners in which the potential of low-stakes assessments for self-regulated learning is realised.

## Introduction

Creating a learning opportunity with the use of assessment, which can benefit self-regulated learning (van der Vleuten et al. 2012), has gained greater prominence in medical education literature (Watling and Ginsburg 2019). It remains a major challenge because assessments designed to inform and support learners’ progress are often perceived to be high-stakes, summative assessments by learners (Bok et al. 2013; Harrison et al. 2016; Heeneman et al. 2015). Utilising the learning potential of assessment requires a better understanding of the mechanisms that explain the relationship between assessment and learning. The current international study involved a focused analysis of data collected as part of a larger research project on the learning potential of programmatic assessment, specifically low-stakes assessments (Schut et al. 2018). One of the findings reported in this previous paper was *that* the teacher–learner relationship was an important factor influencing learners’ perception of assessment stakes. In the study reported in this paper, we explored *how* teacher–learner relationships influence this perception and *how* the influence of these relationships is negotiated in the context of assessment.

Low-stakes assessments are designed to support learning. All kinds of assessment instruments can be used as low-stakes assessments, ranging from multiple choice tests to direct observations of performance in the clinical workplace. They differ from high-stakes assessments in their consequences; low-stakes assessments are feedback-oriented and should hold little consequences to a learner based on their performance. In programmatic assessment they have a dual purpose: as individual assessment to support learning and to provide feedback, and information for learner and teacher, but when aggregated they are used to inform make high-stakes decisions.

Learners’ perceptions of assessment influence how they learn (Ames 1992), and teachers play a powerful role in those perceptions (Schut et al. 2018; Watling and Ginsburg 2019). Teaching happens through human interaction, and therefore the characteristics of teachers’ interaction and relationships with learners can make a substantial difference to the kind of learning environment they create (Haidet and Stein 2006; Ramani et al. 2017; Telio et al. 2015). Research outside the context of medical education has shown that learners’ perceptions of teacher–learner relationships have a large impact on the quality of learners’ motivation, as well as on how they engage in various tasks (den Brok et al. 2004; Wentzel 2003; Wubbels and Brekelmans 2005). Furthermore, learner support has been identified and highlighted as key when we aim to use assessment as a learning opportunity (Eva et al. 2016; Konopasek et al. 2016). However, there is still a significant gap in the medical education literature with respect to the effects of teacher–learner relationships on the use and uptake of assessment information (Haidet and Stein 2006; Telio et al. 2015; Watling and Ginsburg 2019), and therefore the potential of using assessment to support learning. Our aim with this study is to develop a better understanding of how teacher–learner relationships influence the learning potential of low-stakes assessments.

To understand the influence of teacher–learner interactions and relationships on learners’ assessment perception, interpersonal theory is pivotal. The theory states that to describe interpersonal communication, two independent dimensions are both sufficient and necessary: *agency* and *communion* (Bakan 1966; Fournier et al. 2012; Gurtman 2009; Horowitz and Strack 2011). Applied to education (Wubbels et al. 2016), *Teacher Agency* describes the level of teacher influence in the teacher–learner interaction and relationship. *Teacher Communion* refers to the level of warmth or friendliness a teacher communicates in interactions (Gurtman 2009; Pennings et al. 2018; Wiggins 1996; Wubbels et al. 2016).

**Fig. 1:**
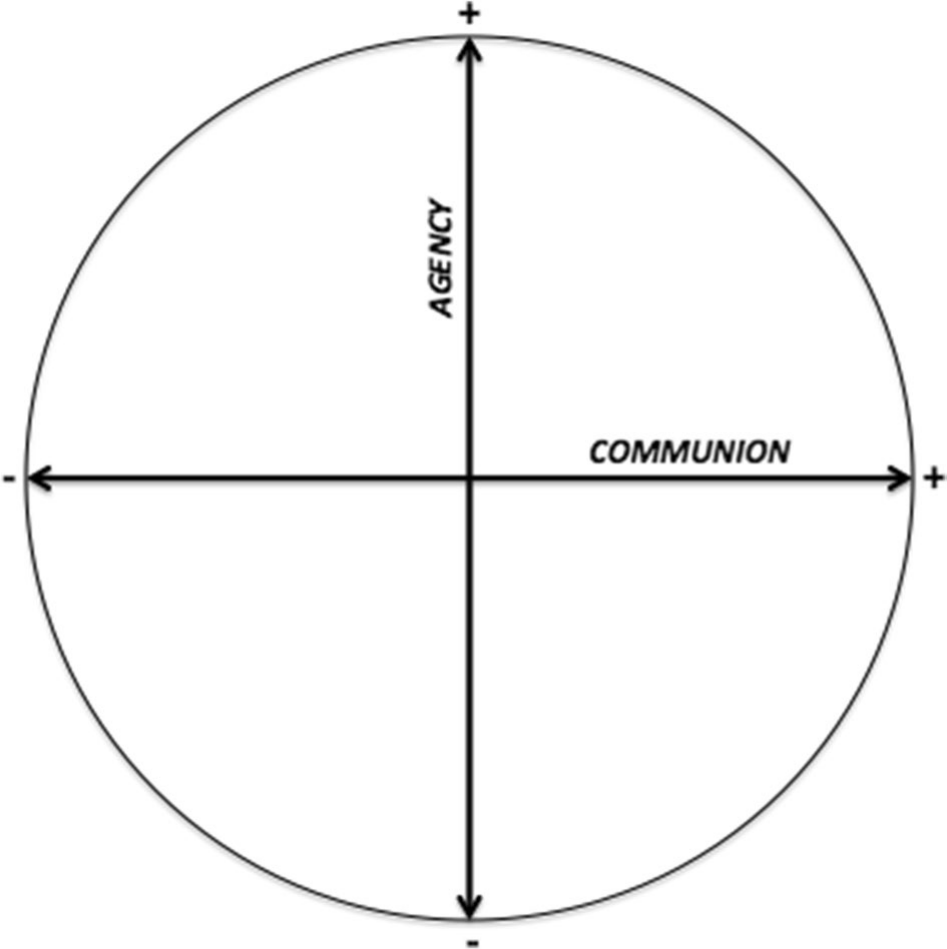
The interpersonal circumplex (e.g. Gurtman 2009; Wiggins 1996)

Interpersonal circumplex models combine these two independent dimensions in one framework, indicating that the interpersonal meaning of behaviour in interaction and positions in relationships can be described as a combination of both dimensions (Fig. 1) (e.g. Gurtman 2009; Wiggins 1996). The principle of complementarity (Horowitz and Strack 2011) suggests that the interpersonal positions of two people in interactions or relationships are not random, but occupy a similar position on the dimension of *communion* (e.g., high teacher communion invites high learner communion and vice versa) and an opposite position of the dimension of *agency* (e.g., high teacher agency will invite low learner agency and vice versa). In the literature, two levels of time are distinguished when describing interpersonal perceptions (Wubbels et al. 2012). Interpersonal perceptions of behaviour and interactions are positioned at the moment-to-moment: the micro-level. Relationships can only be described at a longer period of time: the macro-level. For instance, when a preceptor demands a resident to pay attention during rounds, it is likely that this behaviour is perceived as dominant (micro-level). A physician who has full attention during hand-over will be perceived as having a high agency position in relationship to colleagues (macro-level). Micro and macro-level perceptions mutually influence each other. For instance, supervisors with a high agency position in relationships with residents, will need to ask for attention less often.

Interpersonal theory has been used extensively to investigate teacher–learner interactions and relationships in secondary education (Wubbels and Brekelmans 2005), and in the supervision process within research projects in higher education and PhD projects (de Kleijn et al. 2013; Mainhard et al. 2009). Studies have generally shown positive effects of combinations of teachers’ *agency* and *communion* on cognitive and affective learning outcomes (den Brok et al. 2004; Wentzel 2003; Wubbels and Brekelmans 2005). Teacher support and caring (communion) has been proven to be critically important for learners’ interest in academics (Wentzel 1994), their efficacy and engagement in self-regulated learning, and the decrease of their anxiety about task engagement (Ryan and Patrick 2001). Studies have shown positive influences of teacher communion on feedback receptivity in the context of master thesis supervision (de Kleijn et al. 2013) and PhD supervision (Ives and Rowley 2005). Furthermore, teacher support influences learners’ achievement goals, specifically by promoting the adoption of mastery goals (defined as goals in which the emphasis is on learning, effort, and improvement (Turner et al. 2013)). This is likely to stimulate long-term and high-quality involvement in learning and is associated with a wide range of motivation-related variables that are necessary mediators for self-regulated learning (Ames 1992). Unsurprisingly, learners care about their relationships with teachers and respond with greater engagement and effort when they believe teachers care and are supportive (Urdan and Schoenfelder 2006; Wentzel 1999). Far less attention has been paid to the relation of teachers’ agency with learners’ achievement goals (Mainhard 2015) and assessment task perception, which is of interest in the current study, especially given the hierarchical nature and location of power within any assessment relationship (Heron 1981; Reynolds and Trehan 2000). The context of medical education further complicates the struggle of teachers with how much autonomy to grant learners, given the responsibility and need for high-quality patient care within the medical workplace (Eva et al. 2016; Hauer et al. 2014; Watling and LaDonna 2019).

In the present study, the agency and communion dimensions are used to describe and analyse learners’ reflections on interpersonal relationships with their teachers in the assessment context. The following research question guided the study: How are learners’ perceptions of the interpersonal relationship with teachers in the assessment environment related to learners’ assessment perceptions from early undergraduate medical education to post-graduate training?

## Methods

We used an open and qualitative approach to data gathering and analysis, inspired by constructivist grounded theory (Corbin and Strauss 2008; Watling and Lingard 2012) to explore learners’ experiences with low-stakes assessments in the context of programmatic assessment. Learners’ perceptions of the relationship with their teachers, particularly their sense of agency in this relationship, was identified as a key variable for understanding learners’ assessment perception (Schut et al. 2018). To further delve into this relationship aspect of assessment perception, we turned to interpersonal theory (Horowitz and Strack 2011) where two dimensions used to conceptualise interpersonal perceptions (*agency* and *communion*). These dimensions were used as existing sensitising concepts to help explain the underlying mechanism and enhance our understanding of the influence of interpersonal relationships on the perception and use of low-stakes assessments. We thus used a focussed analysis to examine all data relating to participants’ comments that pertained to their teachers’ interpersonal behaviour or to their interactions or relationships with their teachers, using an interpersonal theory lens to enhance the explanatory power of the emerging theory (Corbin and Strauss 2008). For the purpose of the current study, teachers are defined as ‘all significant others’ whom learners encounter within their assessment experiences. The assessment practices are described in detail elsewhere (Schut et al. 2018).

### Data collection

This study was conducted in three institutes; Cleveland Clinical Lerner College of Medicine (USA), Maastricht University (Netherlands), and Dalhousie University (Canada). The context of medical education varies over time and learners encounter teachers with different educational roles in various assessment environments. Learners move from a highly structured academic learning context in the pre-clinical years, to the more open, and unstructured clinical context of undergraduate and postgraduate medical training. The latter demands a more active, self-steering attitude from learners over time (Deketelaere et al. 2006), which potentially sets different requirements for the relationship with their teachers. We therefore purposefully selected learners experiencing different assessment practices from preclinical and clinical phases: preclinical undergraduate education (setting A, Netherlands and USA); clinical undergraduate education, also known as clerkships (setting B, Netherlands); and post-graduate medical education, also known as residency programs (setting C, Canada, Netherlands). This resulted in a sample from five different assessment programmes from three different institutes. E-mails inviting learners to participate in one-to-one interviews were sent by local faculty members. A convenience sampling approach was taken based on learners’ availability at pre-determined times. A total of 26 learners participated in individual, semi-structured interviews. An overview of participants’ characteristics can be found in Table 1.

**Table 1:** Participants’ characteristics

Characteristics		N=26
*Programme*		
A1	Year 1 & 2 of the 5-year graduate entry programme Physician-Clinical Investigator at Cleveland Clinical Lerner College of Medicine of CWRU, Ohio, USA	5
A2	Year 2 of the 4-year graduate-entry programme Physician-Clinical Investigator at the Faculty of Health, Medicine and Life Science, Maastricht University, the Netherlands	6
B1	The 12 weeks clinical rotation Family Medicine during the undergraduate programme Medicine at Faculty of Health, Medicine and Life Science, Maastricht University, the Netherlands	4
C1	Year 2 of the 2-year Family Medicine residency programme at Dalhousie University Department of Family Medicine, Canada	6
C2	End year 1 and 3 of the 3-year family Medicine residency programme at Maastricht University Medical Centre, Maastricht	5
*Learning and assessment setting*		
	Preclinical training	11
	Clinical training	15
*Gender*		
	Female	18
	Male	8

Open-ended questions were posed by one interviewer (SS), who asked participants to describe (1) their general perceptions of programmatic assessment, (2) their different assessment experiences, (3) how they perceived the assessment stakes and what influenced these stakes. When participants mentioned the role and influence of the teacher, follow-up questions were used to prompt a more detailed reflection on how teachers influenced assessment stakes and how learners perceived the relationship. See Appendix I for the Topic Guide for Interviews. Data were collected between April 2016 and November 2016. Participants received a small compensation (a $/€10 gift card). Ethical approval was obtained from the Dutch Association for Medical Education Ethical Review Board (NVMO-ERB668 on 01/03/2016), the Dalhousie Health Sciences Research Ethics Board (REB#2016-3882 on 25/07/2016) and the Cleveland Clinic Institutional Review Board (IRB#16-1261 on 21/09/2016). The application and approval allowed for the iterative process of data collection and analysis following the open, and explorative nature of our research project. All participants signed the consent form in which they were informed about the nature of our project and analyses, before and after the interview.

### Data analysis

Verbatim transcripts of the interviews were made and analysed using a constant comparison approach, which comprises simultaneous coding and analysis of data in order to develop and refine concepts and explore their inter-relationship (Corbin and Strauss 2008). The focussed analysis combined coding that was guided *a priori* by awareness of interpersonal theory with inductive coding that emerged from the data. Firstly, we identified the points in the transcripts where participants mentioned teachers in their assessment and learning environment. All fragments were re-read to identify whether or not a learner reflected on the behaviour of the teacher, past interpersonal experiences, or the nature of the relationship. These points were coded as ‘Interpersonal Perceptions (IP) fragments’: one or a sequence of sentences relating to the behaviour of the teacher, past interpersonal experiences or the nature of the relationship as perceived by the learner. Secondly, we identified the points in the transcripts where participants mentioned reasons, motivations, goals, and ambitions to engage with their assessment task and how they perceived the assessment task in stakes and learning value. These points were distinguished within the transcripts as learners’ Assessment Task Perception (ATP) fragments. All fragments were re-examined and coded according to the perceived stakes (low- to high-stakes) and the perceived value the assessment has to guide (further) learning. The initial template was used to re-examine all identified fragments and their relationship within each transcript by the primary investigator (SS), allowing additional codes to modify and refine the template. Memos and comments were added to further specify and elaborate code meaning. This helped us understand the richness of the data and the mechanisms involved in interpersonal perceptions, as well as the influence the perceived teacher–learner relationship had on learners’ assessment task perception. A second investigator (SH) independently analysed and re-coded the fragments of the first five interviews. Both investigators then met to compare their interpretations and resolve disagreements through discussion. To further enhance the rigour of the analysis, examples and counter examples were reviewed by a third investigator, who is considered an expert in teacher–learner relationships and teacher education in general (JvT). The final template was discussed with the whole research team and all fragments were re-read (SS) to ensure no relevant information was missed.

We acknowledge that the data as well as the interpretations and meaning we ascribed to these data are co-constructed by interactions with the participants. To prevent biases as much as possible, we collected data from multiple sources and brought together a multidisciplinary research team for analysis: SS and ED have a background in educational sciences; CvdV in psychology, JvT in sociology of education, and SH in biomedical sciences. JvT is not directly involved in medical education. Furthermore, to avoid confirmation bias in our interpretations, ED, SH and CvdV brought an outsider perspective to interpersonal theory.

## Results

Our analysis of the data confirmed that the agency and communion dimensions provide an adequate frame of reference for enhancing our understanding of learners’ perceptions of assessment stakes. The results of these analyses will be described underneath, structured according to the two dimensions, and supported with illustrative examples.

### Accepting teachers’ agency as legitimate

#### Positional agency

The first source of teachers’ agency came with their authority position which is referred to as “positional agency”: agency that stems from holding social positions such as rank or an authority role. Most learners indicated that in the assessment process, they often felt subordinate to teacher’s authority position: they emphasised that teachers had the power to determine content, performance criteria, and consequences, as well as to assess and qualify learners’ performance, based on their formal position as teacher/assessor. Learners especially felt they depended on the teacher as an authority when assessment criteria were perceived as subjective judgments (as contrasted to standardised tests), for example in the case of performance judgements based on work-based observations. Although the assessment context was low-stakes, learners were aware that all encounters could influence or might determine the high-stakes evaluation. Even when the teacher was perceived as supportive, there was still a feeling that “*at the end of the day*” (*C2.1*), it was the decision of the teacher that mattered. Furthermore, there was a general feeling that assessments and evaluations from staff members were valued more highly when evaluating learners’ progress, than for example self- or peer assessments.

It’s known that the assessments from the faculty do carry more weight. Because they come from faculty and their position, I think part of that is just that authority position, that their assessment does carry more weight (A1.12)

Not all positional agency was necessarily accepted as legitimate. In several situations, positional agency led learners to comply with the demands or requirements of the teacher, even when they did not believe these had any value or meaning for them. This tended to alienate them from their learning process and made the whole assessment experience less meaningful for learning.

So that makes me feel overpowered. I think, well, I could explain my point of view, but when he believes it is like that… well I can’t change that […], in the end I’m still dependent on what he believes. (B1.15)

Some learners, especially within undergraduate medical education, were particularly focused on teachers’ positional agency. These learners looked to their teacher for direction, valued teachers’ judgement highly, often wanted to impress teachers, and consequently raised the stakes themselves. They looked for approval and were willing to comply with the normative beliefs of teachers.

The higher up in hierarchy, the higher the stakes become and the more value I attribute to it. If a resident says I’m not doing okay, I think, well, you are just a resident yourself huh, but when a professor says something like that, then I think, oh no. That obviously matters more. (B1.18)

Furthermore, some learners expressed a need for an ‘objective’ qualification of their performance from a formal authority figure for summative, accountability purposes. Although this led to a high-stakes assessment experience, learners could still value the learning opportunity.

#### Expert agency

The second source of teachers’ agency that enhanced teachers’ influence on learners’ assessment perception was expertise. When teachers were perceived as knowledgeable, learners were more willing to accept this base of influence as legitimate and to use assessment as a learning opportunity: *I have grown respect for my particular preceptor, realised over time just how good he is at what he does and so now when he gives me advice, I’m very focused on what that is and how to apply it. (A1.11).* The value of teachers’ expertise did not depend solely on medical expertise, but also on knowledge and experience as an educator. Although teachers’ medical expertise related to the perceived relevance of assessment and learners’ feedback receptivity, trust and safety also related to how knowledgeable the teacher was of the objectives and requirements of medical training and the assessment system.

I think very highly of my preceptor, but as a surgeon that is. If I needed surgery, he would be my go-to. But as a teacher not so much. I don’t care so much what he thinks, he hardly knows what I’m supposed to do or learn, and he has no idea about how I function in practice. He just doesn’t have that expertise. (C2.3).

### Communion: engaging in safe and personal relationships

Recognition and engagement were important for a personal relationship to become established, which contributed to a safe learning environment and the opportunity to use assessment as a learning experience (high communion). Learners seemed more willing to show their weaknesses when they felt that teachers took a genuine interest in their learning process and invested time in understanding and getting to know them. Learners emphasised that sharing personal life experiences with the teacher made them feel the teacher was approachable, which created an assessment environment in which they allowed themselves to be open and to learn from low-stakes assessments.

I think also because you spend so much time together, you see each other the entire day, you go on house visits together, and I, we had lunch with his family at home every day. You just really, you get to know each other. It makes it all feel less high-stake. (B1.15)

In contrast, when learners perceived teachers to be low on communion, they seemed more intent on giving a positive image of themselves and avoiding losing face. When learners encountered teachers who didn’t invest time, didn’t try to get to know them, or showed little interest in their development, assessments rarely led to a learning opportunity. *I think it’s more high*-*stake. The interactions with these staff people, you don’t spend enough time with them, they don’t care, you’re just another learner […], it makes you more nervous, it’s more stressful (C1.8).* Mostly with short-term encounters, and often with preceptors or supervisors from a different rotation or clerkship, learners felt like a burden on the available time of the teacher. They felt less inclined to ask for feedback and generally experienced the assessment as high stakes. Learners indicated that time spent together was an important requirement for an honest and more holistic judgment by the teacher, which they were then more willing to accept.

Yeah, they know my style, they also know my weaknesses and strengths, I think because it’s such a long-term relationship, I feel like it’s easier for me to come to them and say I’m struggling with this. [….] It helps to become more vulnerable and I get to be really honest and look for their guidance. Whereas I might not really do that in any of the other settings because I don’t want to be judged by the preceptors who don’t know me really well, based on one single interaction (C1.8)

### Combining the dimensions: effective assessment relationships to facilitate learning

When learners perceived teachers to be high on agency but not *showing* dominance at the behaviour level, and to be combining it with friendly behaviour (high communion), it was more likely that low-stakes assessment was used as a learning opportunity. These teachers (high agency–high communion) were described as showing a genuine interest in learners’ objectives, strengths and weaknesses. They also allowed learners to influence or determine assessment objectives. In relationships that were characterised by such teacher behaviour, learners felt comfortable and felt more agency in the assessment process. These relationships enabled assessment to be used as a learning opportunity, which is an illustration of the principle of complementarity.

I think it was the best because, she, I felt more that we were like on equal parts […]. So it was that combined discussion and continued learning that was going on. It wasn’t like I am up here on the power differential and talking down to you and this is what you have done, it was working together. (C1.9)

Teachers with high positional agency whose behaviour was not only perceived as dominant but also as uninterested or cold (low communion) caused learners to feel more dependent. In general, these teachers (high agency–low communion) were perceived as less approachable and learners had no influence on the assessment or feedback, making all assessments high-stakes. This translated in the need for teachers’ approval and in less autonomy over their own learning process: “*I will still ask some questions, but it’s more like, gee, just tell me what you want from me.*” (*A2.19*)

## Discussion

In this paper, interpersonal theory provided a useful lens to examine and understand the associations between the characteristics of teacher–learner relationships and learners’ assessment perceptions. Two important sources for teachers’ agency emerged from the data: positional agency and expert agency. When teachers make these sources of agency prominent by behaving dominantly, learners felt subordinate to their teachers, which hindered assessment being used as a learning opportunity. High levels of teacher communion, in particular in relations in which teachers’ agency was exercised less dominant, had a positive impact on the perception of assessment for learning. What this paper adds is a unique understanding of how these relationships influence learners’ assessment experiences, what sources for agency are involved in assessment relationships and how agency is exercised and negotiated in such a context.

The ambitions for self-regulated learning challenge the traditional assessment model in which teachers exercise unilateral intellectual authority over the assessment process (Heron 1981). What counts as legitimate knowledge is in practice often still framed by the teacher and the summative assessment demands (Pryor and Crossouard 2008). This automatically situates the teacher high on agency, which was also found in our study. Competency-based medical education and the use of portfolios, potentially creates even more dependency on teacher’s agency, because such assessments require teachers’ expert judgment (van der Vleuten et al. 2012). Based on our findings, we argue that in order to utilise the learning potential of assessment, the assessment process requires more learner’s agency and a shift to assessment as a process of co-inquiry to determine what counts as legitimate. Teachers with positional and/or expert agency who do not show dominance, and who engage in personal, trusting relationships, are most likely to create safe learning environments in which learners feel they have such agency. Such teachers are able to provide rules and structure when needed, for example when patient safety is at stake, while avoiding restrictions on learner autonomy. As learners proceed through different phases of medical training, the need for rules and structure change, and teachers need to be able to adapt to those needs when striving to benefit self-regulated learning, by entrusting and empowering learners gradually.

The communion dimension and the complementary principle that elicits sameness (e.g., high teacher communion invites high learner communion and vice versa) explained the conditions for a safe and personal relationship to develop, in which assessment could be experienced and used as a learning opportunity. Time was considered an important prerequisite for high-communion relationships to develop, which is also highlighted by other scholars (Ramani et al. 2018; Sargeant et al. 2015; Watling and Ginsburg 2019). Our results showed that during medical training, this is not always possible. Caution should be taken with the influence of short-term interpersonal relationships on learners’ assessment perception. Medical educators should support learners’ resilience for dealing with short-term relationships and the impact these have on assessment and learning and create awareness amongst teachers that assessment should be approached with more caution when a relationship is lacking.

Practical implications of this study appertain to teacher professionalization. We argue it is worthwhile to consider how learners perceive teachers interpersonally when one is aiming to use assessment to create a learning opportunity. The ‘Interpersonal Circumplex” (Gurtman 2009) was found useful to understand the influence of teacher–learner relationships on learners’ assessment perception, and which type of interactions led learners to use the assessment experience as a learning opportunity. Given that interactions are seen as the building blocks of relationships (Fournier et al. 2012; Pennings et al. 2018; Wubbels and Brekelmans 2005), knowledge of those interactions and the influences these have on the use and uptake of assessment, are useful when implementing the use of assessment for learning. This is particularly the case for the diagnosis of (problematic) interactions or undesirable assessment perceptions, but also for the design of effective interventions that stimulate a more effective relationship between teachers and learning in the context of assessment. We argue that the Interpersonal Circumplex (Gurtman 2009) could be used to create awareness amongst teachers on how they influence learners’ assessment experiences and how they can alter these interactions and engage in effective relationships, as has been done in the domain of teacher education (van Tartwijk et al. 2011). The situational context of medical education and assessment is important here. Medicine’s learning culture might constrain its own teachers for building relationships that make meaningful contributions to learners’ development, due to the important values of independence and autonomy that characterise medicine’s culture (Eva et al. 2016; Harrison and Wass 2016; Ramani et al. 2017; Shepard 2016). Furthermore, we acknowledge that teachers might face significant dilemmas and struggle with the tension that exists between their supportive role in monitoring and facilitating learners’ development and their judgmental responsibility as assessor of learners’ performance and achievement (Rea-Dickins 2004; Watling 2016; Wiliam 2011), especially given the requirements of their role within medical education to warrant the quality of future doctors, who will be required to provide safe and effective health care (Eva et al. 2016).

### Limitations

This study has several limitations. Firstly, the approach adopted here is that all analyses are based on learners’ perceptions only. Although many studies have advocated that how learners perceive teachers’ behaviour may be more informative than more objective measures (Shuell 2010), this could have influenced our results. High correspondence between different respondents’ perceptions of the same type of interactions leading to the same assessment perceptions did strengthen our interpretations and conclusions of the value of the interpersonal dimensions to explain, understand and eventually steer assessment perceptions and the utilising of the potential of programmatic assessment. We do stress the importance of including teachers’ perceptions and observations of interpersonal communication to further build this theory. Secondly, learners participated voluntarily which potentially creates a selection bias. It might very well be that these learners’ reflections might not represent those of other learners. Finally, the data were collected within different institutes and phases of medical education, limiting the number of interviews per setting. Especially given the cultural nature of concepts like interpersonal relationships, assessment, and self-regulated learning, we stress the importance of further testing the value of interpersonal theory within different disciplines of medical education in various countries. Furthermore, a study using a longitudinal design could shed more light on the development of learners’ perception of teacher–learner relationships in the context of assessment.

## Conclusion

The ‘Interpersonal Circumplex’ (Gurtman 2009) was found useful to understand the influence of teacher–learner relationships on learners’ assessment perception, and which type of interactions led learners to use the assessment experience as a learning opportunity. We argue that although teacher–learner relationships in the context of assessment are essentially unequal, their purpose should be to reduce this inequality when one is aiming to create an assessment environment that facilitates self-regulated learning. Ideally, the teacher would help learners’ competency development, and their ability to set and achieve useful goals, without trying to oppose or override learners’ agency. Medical education might still be far from establishing a true learning culture (Konopasek et al. 2016; Watling and Ginsburg 2019), but the Interpersonal Circumplex model could be instrumental to help us to move towards a culture that emphasises learning and that supports continuous improvement. Faculty development focussing on interpersonal relationships should be given priority, given the challenges teachers face in overcoming traditional power relationships in assessment and their responsibly to patient safety within the context of medicine specifically. However, teachers have the power to enable the use of assessment as a learning opportunity and should receive support in fulfilling that promise.

## Appendix: Topic guide for interviews

**Topics and promoting questions**

1. **Low-stake assessment**
  - Different low-stake assessment experience (if needed in comparison to high-stake assessment)
  - Information and feedback fostering all learning from assessment
  - *Role of the teacher, assessor, supervisor*

**HOW—WHY—EXAMPLES**

General prompting questions

- *Can you give an example? Can you give a counter example?*
- *Can you elaborate more on this?*
- *Why is that?*
- *Can you explain this?*
- *How do you feel about this?*

*Topic A*

- *Could you describe the stakes you perceive in that assessment? Do you have other experiences?*
- *What influenced the stakes involved?*
- *Does this apply for all assessment experiences (good, bad, low*-*stakes, high*-*stakes, positive, negative, etc.)*
- *Could you describe the consequences related to your performance on the assessment task?*
- *Can you tell us about the emotions related to these consequences you experienced?*
- *How did you feel about these consequences?*
- *What or who decided on these consequences?*

*Topic B*

- *Could you tell us something about the information you gain from assessment tasks that benefit your learning? Do you have counter examples?*
- *Could you describe assessment tasks or learning activities that provided you with information concerning you progress or development? Do you have counter examples?*
- *Why/how did you use this information? Do you have counter examples?*
- *Did you have control about assessment, feedback, time, content, etc.?*

*Topic C*

- *How would you describe this relationship with your assessor/teacher/supervisor?*
- *How do you experience this relationship?*
- *Can you describe the role of your teacher, assessor, supervisor, etc. more elaborately? Do you experience any differences?*
- *How long did this relationship last? Did that influence the assessment experience or your relationship? Could you describe how? Do you have counter examples?*
- *What does this mean for the assessment experience and stakes involved?*
- *Are there other examples of relationships that influenced your assessment experiences?*

2. **Best-practices of assessment and information fostering learning**

**HOW—WHY—EXAMPLES**

Prompting questions

- *What do you consider best*-*practices with low*-*stake assessment?*
- *What made them a best practice?*
- *What were the consequences of this assessment?*
- *How did you feel about these consequences?*

3. **Room for not-discussed themes**
